# Effects of Nitrite Stress on the Antioxidant, Immunity, Energy Metabolism, and Microbial Community Status in the Intestine of *Litopenaeus vannamei*

**DOI:** 10.3390/antiox13111318

**Published:** 2024-10-29

**Authors:** Yafei Duan, Guowei Zhong, Yuxiu Nan, Yukai Yang, Meng Xiao, Hua Li

**Affiliations:** 1 Key Laboratory of South China Sea Fishery Resources Exploitation & Utilization, Ministry of Agriculture and Rural Affairs, State Key Laboratory of Mariculture Biobreeding and Sustainable Goods, South China Sea Fisheries Research Institute, Chinese Academy of Fishery Sciences, Guangzhou 510300, China; 2 Key Laboratory of Efficient Utilization and Processing of Marine Fishery Resources of Hainan Province, Sanya Tropical Fisheries Research Institute, Sanya 572018, China

**Keywords:** shrimp, nitrite, intestine, physiological response, microbial community

## Abstract

Nitrite is the main environmental pollutant that endangers shrimp culture. Intestinal health is essential for the disease resistance of shrimp. In this study, *Litopenaeus vannamei* shrimps were separately exposed to 1 and 5 mg/L of nitrite stress for 48 h, and then the variations in intestinal health were investigated from the aspects of histology, antioxidant, immunity, energy metabolism, and microbial community status. The results showed that nitrite stress damaged intestinal mucosa, and 5 mg/L of nitrite induced more obvious physiological changes than 1 mg/L. Specifically, the relative expression levels of antioxidant (*ROMO1*, *Nrf2*, *SOD*, *GPx,* and *HSP70*), ER stress (*Bip* and *XBP1*), immunity (*proPO*, *Crus*, *ALF*, and *Lys*), inflammation (*JNK* and *TNF-α*), and apoptosis (*Casp-3* and *Casp-9*) genes were increased. Additionally, intestinal energy metabolism was activated by inducing glucose metabolism (*HK*, *PK*, *PDH*, and *LDH*), lipid metabolism (*AMPK* and *FAS*), tricarboxylic acid cycle (*MDH*, *CS*, *IDH*, *SDH*, and *FH*), and electron transfer chain (*NDH*, *CytC*, *COI*, *CCO,* and *AtpH*) gene transcription. Further, the homeostasis of intestinal microbiota composition was also disturbed, especially the abundance of some beneficial genera (*Clostridium sensu stricto 1*, *Faecalibacterium*, *Romboutsia*, and *Ruminococcaceae UCG-010*). These results reveal that nitrite stress could damage the intestinal health of *L. vannamei* by destroying mucosal integrity, inducing oxidation and ER stress, interfering with physiological homeostasis and energy metabolism, and disrupting the microbial community.

## 1. Introduction

Pacific white shrimp *Litopenaeus vannamei* is the main economic shrimp species worldwide. Disease is still the main reason hindering the development of the shrimp industry, with environmental stress being the key inducer [1,2]. Nitrite is the main harmful environmental pollutant in shrimp culture, which is an intermediate product in the conversion of ammonia from nitrogen-containing wastes to nitrates [3]. Nitrite-N concentration in shrimp cultures can increase above 5 mg/L [4] and reach as high as 20 mg/L [3]. Nitrite is a highly toxic substance, which seriously affects the growth and survival of shrimp and leads to frequent diseases [5,6,7,8]. For example, nitrite stress can affect the formation of hemocyanin in shrimp, lead to hypoxia in tissues, and then damage the respiratory metabolism and immune system [6]. Oxidative stress induced by nitrite increases the risk of hepatopancreatic cell apoptosis [9]. Nitrite stress can cause pathological changes in the shrimp hepatopancreas, resulting in autophagy and apoptosis [10]. Nitrite stress interferes with the acid–base balance of hemolymph and induces electrolyte changes in *Marsupenaeus japonicus* [11]. A high concentration of nitrite (46.2–138.6 mg/L) interferes with the energy metabolism homeostasis of *L. vannamei* [12]. Nitrite stress can also induce the levels of glucose and lactic acid in the hemolymph of *L. vannamei* [13]. Therefore, exploring the physiological response characteristics of shrimp to nitrite stress is conducive to the establishment of relevant antistress approaches.

The intestine is the organ responsible for nutrient metabolism and immunity in shrimp and also the main place where diseases occur [12,13,14,15,16,17]. Intestinal health is beneficial to improve the immunity and antistress ability of shrimp, while intestinal injury causes digestive dysfunction and increases susceptibility to diseases. Intestinal health depends on its barrier function, which is mainly composed of the intestinal mucosa, immune components, and microbial communities [18]. A stable intestinal microbiota is beneficial to the physiological stability of the host, while an unbalanced microbiota will aggravate the physiological disorder of the host [15,19]. Previous studies have shown that 20 mg/L of acute nitrite stress induced intestinal antioxidant index disturbance and disturbed the intestinal microbiota of *L. vannamei* [18,20]; 10 mg/L of acute nitrite stress can alter the intestinal microbial community of *L. vannamei* by interfering with host–microbiota interactions [21]. Additionally, chronic nitrite stress can also reduce the immunity of *L. vannamei* by disrupting the homeostasis of the intestinal microbial community [7]. Although there is some exploration of the toxicity of nitrite stress in shrimp, comprehensive study insights into intestinal health are limited.

Therefore, in this study, we selected 1 and 5 mg/L of nitrite, which are common concentrations in shrimp cultures, to investigate how they affect the intestinal health of *L. vannamei* from different perspectives of intestinal barrier function. The changes in the histomorphological, stress response, immunity, energy metabolism, and microbial community status were explored. Finally, a potential mechanism for the adverse effects of nitrite stress on the intestinal health of shrimp was also derived. These results can provide new insights into the toxicity of nitrite stress to the shrimp intestine.

## 2. Materials and Methods

### 2.1. Temporary Rearing of the Shrimp

The healthy *L. vannamei* shrimp used in this study were not in the molting stage, and no molting occurred throughout the experiment. The shrimp were sourced from local intensive shrimp ponds in Shenzhen (China), with an average body weight of 4.6 ± 0.2 g. Prior to the nitrite stress exposure experiment, all shrimp were temporarily cultured in aquarium tank for 3 days, with continuous aeration for 24 h. The water quality met the requirements of shrimp culture: a water temperature of 29 °C, a pH of 7.9–8.0, and salinity of 30‰. The water was changed daily. The shrimp were fed a compound feed at a rate of 5% of their body weight every day, with adjustments made based on feeding observations. Feces and leftover feed were promptly removed.

### 2.2. Nitrite Stress Exposure and Sampling

After three days of temporary culture, we randomly divided the shrimp into three groups: control (CK), 1 mg/L nitrite-N stress (N1), and 5 mg/L nitrite-N stress (N5). Each group consisted of three tanks, with 50 shrimp per tank. The CK group was maintained in normal filtered seawater without sodium nitrite. The nitrite-N concentrations in the rearing water for N1 and N5 groups were set at 1 mg/L and 5 mg/L, respectively, which was achieved by adding sodium nitrite. During the stress exposure experiment, water was changed daily and sodium nitrite was added to maintain the target concentrations. Aside from differing nitrite-N levels, all other culture conditions remained consistent across the three groups during the temporary culture period. The stress exposure experiment lasted for 48 h.

Samples were collected 48 h post-stress induction. From each tank, the middle intestines of three shrimp were sampled for histopathological analysis; the whole intestines of five shrimp were sampled, mixed, and placed in RNAFollow solution (NCM Biotech, Suzhou, China) at 4 °C for 24 h and then stored at −80 °C for gene expression analysis. Additionally, the whole intestines of 10 shrimp were pooled for microbial community analysis.

### 2.3. Histomorphological Analysis

Shrimp intestines were fixed in 4% paraformaldehyde for 24 h. After rinsing with flowing water for 30 min, the tissues were dehydrated in a series of ethanol solutions (70%, 80%, 90%, and 100%). They were then made transparent with xylene, embedded in paraffin, and 4 μm thick sections were prepared using a microtome (Leica RM2016, Shanghai, China). Following hematoxylin-eosin (HE) staining, the stained sections were examined and photographed under a microscope (Nikon, Tokyo, Japan).

### 2.4. Gene Expression Analysis

Total RNA was extracted from the intestines of the shrimp using TRIzol reagent, and genomic DNA was removed using DNase. The quality and concentration of RNA were determined using a nucleic acid quantification instrument and 1.0% agarose gel electrophoresis, respectively. The purified RNA was reverse transcribed into cDNA using Servicebio RT First Strand cDNA Synthesis Kit (Wuhan, China) and stored at −20 °C until analysis.

Real-time quantitative PCR (qPCR) was used to analyze the expression of target genes using the SGExcel Fast SYBR qPCR Mixture Kit (Sangon Biotech, Shanghai, China) on a real-time PCR system (CG-05, Heal Force, Shanghai, China). The *β-actin* gene served as a reference gene, and the specific qPCR primer sequences are shown in Table S1. The qPCR reaction had a total volume of 15 μL, comprising 7.5 μL SYBR Green Pro Taq HS Premix (2×), 1.0 μL cDNA, 0.6 μL forward primer (10 μmol/L), 0.6 μL reverse primer (10 μmol/L), and 5.3 μL RNASe-free water. The qPCR cycling conditions were 95 °C for 30 s, followed by 40 cycles of 95 °C for 5 s and 60 °C for 30 s. The relative mRNA expression levels of the target genes were calculated using the Livak and Schmittgen method (2001) [22], expressed as the fold relative to the CK group.

### 2.5. Intestinal Microbial Community Analysis

We extracted the total genomic DNA from intestinal microbial samples using the Fast DNA SPIN kit for feces (MPBIO, Santa Ana, CA, USA) according to the manufacturer’s instructions. The concentration and purity of the extracted DNA were analyzed using 1% agarose gel electrophoresis. A pair of barcoded primers was used to amplify the V4 region of the 16S rDNA genes, specifically the primers 515F and 806R with the barcode. The PCRs were carried out in a total volume of 30 μL containing 10 ng of template DNA, 15 μL of Phusion^®^ High-Fidelity PCR Master Mix (New England Biolabs, Ipswich, MA, USA), and 0.2 μM of both forward and reverse primers. The PCR program consisted of 1 cycle of 98 °C for 1 min, followed by 30 cycles of 98 °C for 10 s, 50 °C for 30 s, and 72 °C for 1 min, finishing with an extension at 72 °C for 5 min. PCR products were detected via 2.0% agarose gel electrophoresis, and samples exhibiting a bright main band between 400 and 450 bp were selected for further experiments. The selected PCR products were mixed in equal ratios and purified using the GeneJET Gel Extraction Kit (Thermo Scientific, Shanghai, China). Sequencing libraries were constructed using the NEB Next^®^ Ultra™ DNA Library Prep Kit for Illumina (New England Biolabs, Ipswich, MA, USA) according to the manufacturer’s protocol, with index codes added. The quality of the library was assessed using a Qubit@ 2.0 Fluorometer (Thermo Scientific, Shanghai, China) and an Agilent Bioanalyzer 2100 system. Finally, the library was sequenced on an Illumina Novoseq PE250 platform, generating paired-end reads.

Paired-end reads were merged using FLASH and assigned to each sample based on unique barcodes. Sequence analysis was performed with UPARSE v10 software, defining operational taxonomic units (OTUs) with ≥97% similarity. Chimeric sequences were identified using UCHIME software. Alpha diversity was calculated based on four metrics: ACE, Chao1, Simpson, and Shannon indices, employing Mothur v1.30.1 software. Rarefaction curves were generated based on these metrics. A Venn diagram was created to quantify the number of unique and shared OTUs across multiple samples. A Principal Component Analysis (PCA) was performed to evaluate beta diversity. A bar plot of the microbial communities was constructed at the phylum level and a heatmap was generated using the heatmap 2 function of the R g-plot package, focusing on the top 50 bacterial genera. Linear discriminant analysis (LDA) effect size (LEfSe) analysis was conducted for quantitative analysis of biomarkers within different groups using the Python LEfSe package. Correlation networks of intestinal bacteria were constructed based on the abundance profiles of individual OTUs using Cytoscape software (http://www.cytoscape.org/, accessed on 28 June 2024). Random forest analyses were performed to analyze metabolic pathway changes according to the Kyoto Encyclopedia of Genes and Genomes (KEGG) catalogue using the R randomForest package.

### 2.6. Statistical Analysis

All biochemical and gene index data are expressed as mean ± standard error (SE), and statistical analysis was performed using one-way analysis of variance using SPSS 27.0 software. A *p* < 0.05 was considered indicative of a significant difference.

## 3. Results

### 3.1. Intestinal Histomorphological Changes

**Figure 1:**
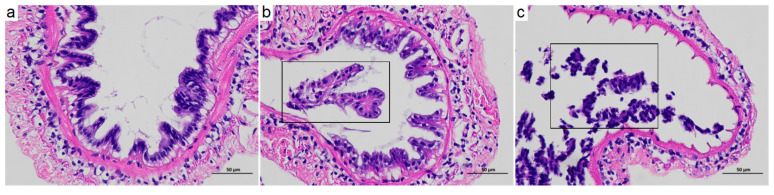
Changes in the intestinal tissue morphology of *L. vannamei* after acute nitrite stress. (**a**) CK group; (**b**) N1 group; (**c**) N5 group. 400× magnification. The black boxes indicate damaged and shed mucosal epithelial cells.

Morphological changes in the intestinal tissue were analyzed using HE staining. In the CK group, the intestinal tissue morphology was relatively intact, with the mucosa connected to the inner wall of the intestine (Figure 1a). Following nitrite stress, the intestinal mucosa of the N1 group showed partial exfoliation (Figure 1b). With increasing exposure concentration, the degree of intestinal mucosal exfoliation in the N5 group increased (Figure 1c).

### 3.2. Antioxidant and ER Stress Responses

**Figure 2:**
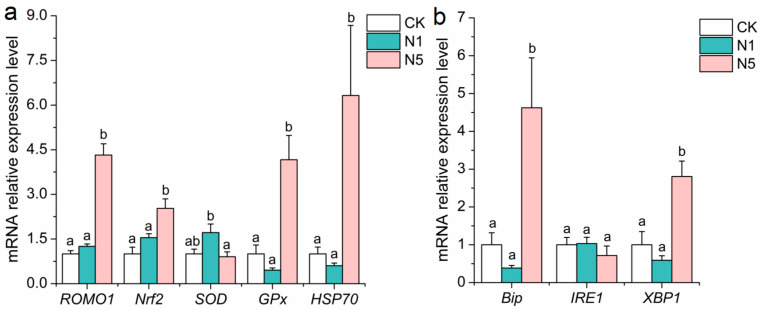
Changes in the expression of redox and endoplasmic reticulum (ER) stress-related genes in the intestinal tissue of *L. vannamei* after acute nitrite stress. (**a**) Antioxidant-related and (**b**) ER stress-related genes. The bars represent the mean ± SE (*n* = 3). The lowercase letters (a,b) on the bars indicate significant differences (*p* < 0.05) between the groups.

Compared with the CK group, the relative expression levels of antioxidant signaling genes, such as reactive oxygen species regulator 1 (*ROMO1*), nuclear factor erythroid-derived 2-like 2 (*Nrf2*), glutathione peroxidase (*GPx*), and heat shock protein 70 (*HSP70*) were significantly up-regulated in the N5 group. In contrast, these genes did not show significant changes in the N1 group. The relative expression levels of superoxide dismutase (*SOD*) were up-regulated in the N1 group; however, this change was not statistically significant (*p* > 0.05) (Figure 2a). Endoplasmic reticulum (ER) stress-related genes, such as immunoglobulin binding protein (*Bip*) and X-frame binding protein 1 (*XBP1*), were significantly up-regulated in the N5 group (*p* < 0.05), whereas no significant changes were observed in the N1 group (*p* > 0.05). The level of inositol-requiring enzyme 1α (*IRE1*) showed no significant change in either the N1 or N5 groups (*p* > 0.05) (Figure 2b).

### 3.3. Changes in the Expression of Physiological-Related Genes 

**Figure 3:**
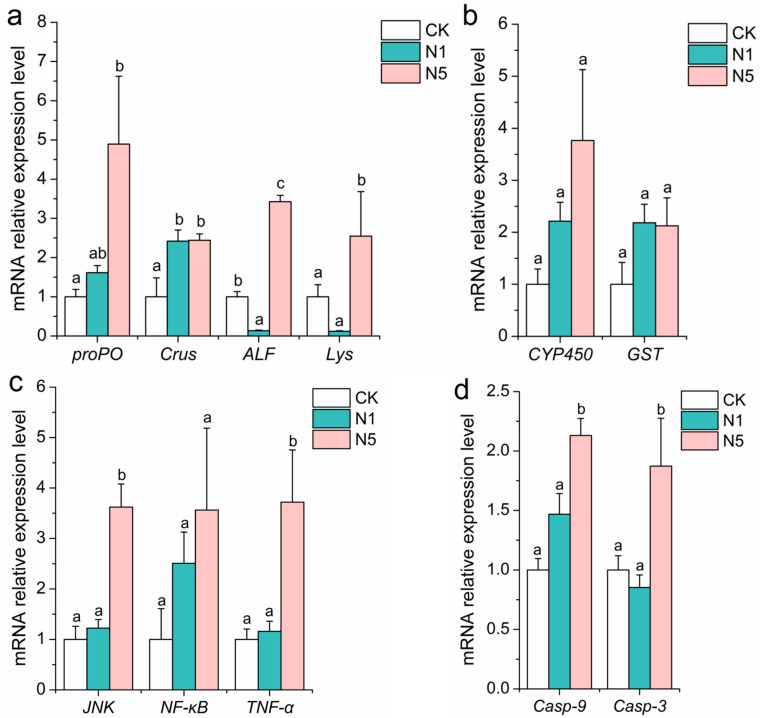
Changes in the expression of physiology-related genes in the intestinal tissue of *L. vannamei* after acute nitrite stress. (**a**) Immune-related; (**b**) detoxification-related; (**c**) inflammation-related; and (**d**) apoptosis-related genes. The bars represent the mean ± SE (*n* = 3). The lowercase letters (a–c) on the bar indicate significant differences (*p* < 0.05) between the groups.

Compared with the CK group, the relative expression levels of antimicrobial genes, such as prophenoloxidase (*proPO*) and crustin (*Crus*), were up-regulated in the N1 group, whereas anti-lipopolysaccharide factor (*ALF*) and lysozyme (*Lys*) were down-regulated; however, the changes in *proPO* and *Lys* in the N1 group were not significant (*p* > 0.05). Whereas the levels of *proPO*, *Crus*, *ALF,* and *Lys* were significantly up-regulated in the N5 group (*p* < 0.05) (Figure 3a). Detoxification-related genes, such as cytochrome P450 (*CYP450*) and glutathione S-transferase (*GST*), showed increased relative expression levels in both N1 and N5 groups; however, these differences were not statistically significant (*p* > 0.05) (Figure 3b). Inflammation-related genes, such as c-Jun amino-terminal kinase (*JNK*) and tumor necrosis factor-α (*TNF-α*), had significantly up-regulated relative expression levels in the N5 group (*p* < 0.05), whereas no significant changes were observed in the N1 group (*p* > 0.05). The level of nuclear factor kappa-B (*NF-κB*) gene was up-regulated in the N1 and N5 groups; however, the differences were not significant (*p* > 0.05) (Figure 3c). For apoptosis-related genes, such as caspase-3 (*Casp-3*) and caspase-9 (*Casp-9*), relative expression levels were significantly up-regulated in the N5 group (*p* < 0.05). Although the level of *Casp-9* was slightly up-regulated in the N1 group, this change was not significant (*p* > 0.05) (Figure 3d).

### 3.4. Changes in the Expression of Energy Metabolism-Related Genes 

#### 3.4.1. Carbohydrate Metabolism

**Figure 4:**
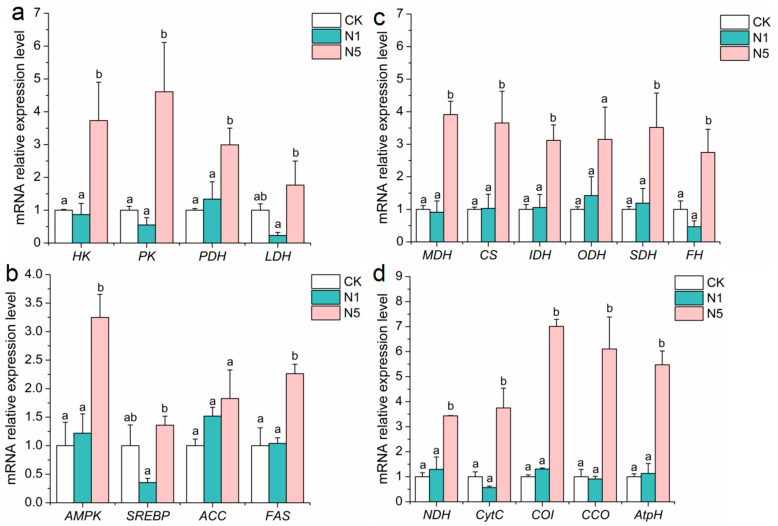
Changes in the expression of energy metabolism-related genes in the intestinal tissue of *L. vannamei* after acute nitrite stress. (**a**) Carbohydrate-metabolism-related; (**b**) lipid-metabolism-related; (**c**) tricarboxylic-acid-cycle-related; and (**d**) electron transport-chain-related genes. The bars represent the mean ± SE (*n* = 3). The lowercase letters (a,b) on the bar indicate significant differences (*p* < 0.05) between the groups.

Compared to the CK group, the relative expression levels of carbohydrate-metabolism-related genes, such as hexokinase (*HK*), pyruvate kinase (*PK*), pyruvate dehydrogenase (*PDH*), and lactate dehydrogenase (*LDH*), were significantly up-regulated in the N5 group (*p* < 0.05). In contrast, no significant changes were observed in the N1 group (*p* > 0.05) (Figure 4a).

#### 3.4.2. Lipid Metabolism

Compared to the CK group, the relative expression levels of lipid-metabolism-related genes, such as AMP-activated protein kinase (*AMPK*) and fatty acid synthase (*FAS*), showed no significant changes in the N1 group (*p* > 0.05) but were significantly up-regulated in the N5 group (*p* < 0.05). In contrast, the levels of cholesterol regulatory element binding protein (*SREBP*) and acetyl-CoA carboxylase (*ACC*) showed no significant changes in either the N1 or N5 groups (*p* > 0.05) (Figure 4b).

#### 3.4.3. Tricarboxylic Acid (TCA) Cycle

Compared to the CK group, the relative expression levels of TCA-cycle-related genes, such as malate dehydrogenase (*MDH*), citrate synthase 1 (*CS*), isocitrate dehydrogenase (*IDH*), succinate dehydrogenase (*SDH*), and fumarase (*FH*), showed no significant changes in the N1 group (*p* > 0.05) but were significantly up-regulated in the N5 group (*p* < 0.05). The levels of oxoglutarate dehydrogenase (*ODH*) were slightly up-regulated in both the N1 and N5 groups; however, this change was not statistically significant (*p* > 0.05) (Figure 4c).

#### 3.4.4. Electron Transfer Chain

Compared to the CK group, the relative expression levels of electron transfer-chain-related genes, such as NADH dehydrogenase (*NDH*), cytochrome c (*CytC*), cytochrome oxidase I (*COI*), cytochrome c oxidase (*CCO*), and ATP synthase (*AtpH*), showed no significant changes in the N1 group (*p* > 0.05); however, they were significantly up-regulated in the N5 group (*p* < 0.05) (Figure 4d).

### 3.5. Changes in Intestinal Microbial Community

#### 3.5.1. Intestinal Microbial Diversity Alterations

**Figure 5:**
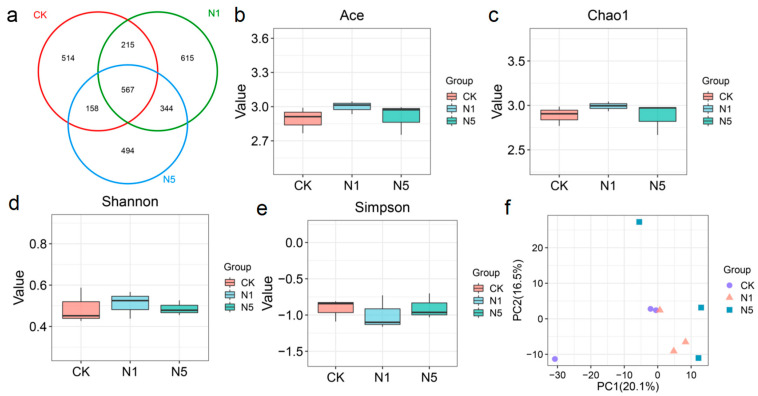
Changes in intestinal microbial diversity of *L. vannamei* after acute nitrite stress. (**a**) The Venn analysis of the microbial OTUs, and (**b**) ACE, (**c**) Chao1, (**d**) Shannon, and (**e**) Simpson indices; (**f**) the *β*-diversity of microbial community based on the PCA plot.

Changes in intestinal microbiota were analyzed using 16S rRNA high-throughput sequencing. The three groups collectively comprised 567 OTUs, with the highest number of unique OTUs found in the N1 group, followed by the CK and N5 groups (Figure 5a). Compared to the CK group, α-diversity indices of intestinal microbiota, such as ACE, Chao1, and Shannon, showed slight increases in the N1 and N5 groups, whereas Simpson indice showed slight decrease in the N1 group but no obvious change in the N5 group; however, none of these indices were statistically significant (Figure 5b–e). Based on the PCA plot, the microbial community pattern of the CK group could not be clearly separated from that of the N1 group; however, it could be distinctly distinguished from that of the N5 group (Figure 5f). However, no significant differences were observed between them based on PERMANOVA analysis (*p* > 0.05) (Table S2).

#### 3.5.2. Changes in Intestinal Bacterial Composition 

**Figure 6:**
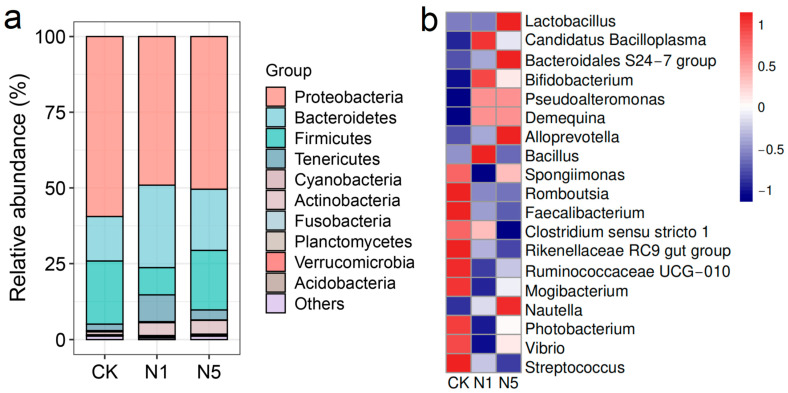
Changes in the composition of intestinal microbial community of *L. vannamei* after acute nitrite stress. (**a**) Relative abundance of bacterial phyla and (**b**) relative abundance of bacterial genera.

The abundance of intestinal bacteria varied at different taxonomic levels. Compared to the CK group, the relative abundance of the phyla Bacteroidetes, Tenericutes, and Actinobacteria increased in the N1 and N5 groups, whereas the relative abundance of Proteobacteria decreased; the abundance of Firmicutes decreased in the N1 group (Figure 6a). However, no significant differences (*p* > 0.05) were observed in the relative abundances of these phyla (Table S3).

Furthermore, the relative abundances of genera such as *Rikenellaceae RC9 gut group*, *Ruminococcaceae UCG-010*, and *Mogibacterium* were significantly decreased in the N1 and N5 groups (*p* < 0.05); *Bacillus* was significantly increased only in the N1 group (*p* < 0.05). Additionally, the relative abundances of *Lactobacillus*, *Candidatus Bacilloplasma*, *Bacteroidales S24-7 group*, *Bifidobacterium*, *Pseudoalteromonas*, *Demequina*, *Alloprevotella*, and *Nautella* increased in the N1 and N5 groups, whereas those of *Spongiimonas*, *Romboutsia*, *Faecalibacterium*, *Clostridium sensu stricto 1*, *Photobacterium*, *Vibrio*, and *Streptococcus* decreased, although no significant differences were observed (*p* > 0.05) (Figure 6b, Table S4).

#### 3.5.3. Changes in Intestinal Microbial Phenotypes 

**Figure 7:**
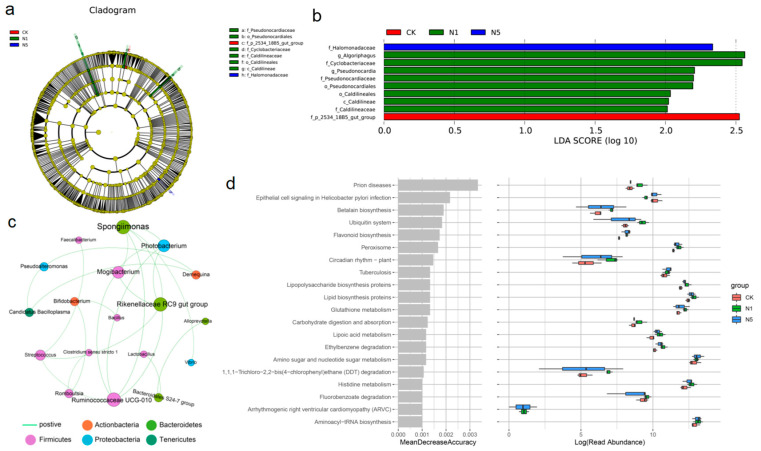
Changes in the phenotypic characteristics of intestinal microbial communities of *L. vannamei* after acute nitrite stress. (**a**) LEfSe cladogram. (**b**) LDA score. (**c**) Correlation network of bacterial genera. The size of the nodes indicates their preponderance in the community. (**d**) Prediction of microbiota function based on the top 20 pathways.

Differential bacterial taxa associated with stress exposure were analyzed. Based on the cladogram and LDA scores > 2.0, the family p_2534_18B5_gut_group was dominant in the CK group, whereas the families Pseudonocardiaceae, Cyclobacteriaceae and Caldilineaceae, along with the genera *Algoriphagus* and *Pseudonocardia* were dominant in the N1 group. Additionally, the family Halomonadaceae was dominant in the N5 group (Figure 7a,b).

The relationship network between intestinal bacteria was analyzed. The phylum Planctomycetes was positively correlated with Tenericutes but negatively correlated with Firmicutes; Fusobacteria was positively correlated with Verrucomicrobia (Figure S1). The genera *Spongiimonas*, *Rikenellaceae RC9 gut group*, *Ruminococcaceae UCG-010*, and *Mogibacterium* were positively correlated with *Photobacterium*. Additionally, *Spongiimonas* was positively correlated with *Vibrio*, whereas, *Faecalibacterium*, *Rikenellaceae RC9 gut group*, and *Ruminococcaceae UCG-010* were positively correlated with *Streptococcus* (Figure 7c).

#### 3.5.4. Changes in Intestinal Microbial Metabolic Function 

Changes in the metabolic functions of the intestinal microbiota were predicted. Compared to the CK group, the functions of “amino sugar and nucleotide sugar metabolism”, “histidine metabolism”, “lipoic acid metabolism”, “flavonoid biosynthesis”, “lipid biosynthesis proteins”, and “lipopolysaccharide biosynthesis proteins” were enhanced in the N1 and N5 groups; however, these changes were not statistically significant (Figure 7d).

## 4. Discussion

Shrimp cultures have long been affected by nitrite toxicity. The intestinal health of shrimps directly affect their physiological homeostasis and antistress abilities. In the present study, nitrite stress caused damage and shedding of the intestinal mucosa of shrimp, which inevitably affected their physiological stability.

### 4.1. Intestinal Stress Response of Shrimps to Nitrite Stress

Oxidative stress is a toxic effect of nitrite in aquatic animals. ROMO1 induces ROS production in cells [23]. GPx is an important antioxidant enzyme that protects cells from oxidative stress [6]. Nrf2 is a transcription factor that induces the expression of antioxidant enzyme genes, thereby protecting organisms from oxidative stress [24]. Heat shock protein 70 is an important stress-related protein that plays an antioxidant role [25]. Notably, 20 mg/L nitrite has been shown to induce oxidative stress and disturb the antioxidant status of intestines of *L. vannamei* [20]. Similarly, in this study, the expression of *ROMO1*, *Nrf2*, *GPx,* and *HSP70* was up-regulated after exposure to 5 mg/L nitrite stress, indicating that the shrimp intestine employs *Nrf2*/*GPx* signaling and stress proteins to respond to oxidative stress. Environmental stress can also cause ER stress, with Bip, IRE1, and XBP1 acting as stress markers [26]. In this study, 5 mg/L nitrite stress also induced the up-regulation of *Bip* and *XBP1* gene expression, indicating that ER stress occurs in the intestine via *Bip*/*XBP1* signaling.

### 4.2. Intestinal Immune Response of Shrimps to Nitrite Stress

Intestinal immunity is an important defense mechanism against stress in shrimps. Nitrite stress affects the expression of immune-related genes in the hepatopancreas of *M. japonicus* in a dose- and time-dependent manner [27]. The proPO system and antimicrobial molecules, such as Crus, ALF, and Lys, are the main components of nonspecific immunity in shrimps [28,29]. In this study, changes in the expression of *proPO*, *Crus*, *ALF,* and *Lys* genes indicated that nitrite stress disturbed the intestinal immune homeostasis in shrimps. NF-κB can activate inflammatory factors such as TNF-α [30]. JNK plays an important role in linking inflammation and apoptosis [31]. Apoptosis is beneficial for maintaining homeostasis in the internal environment and caspase family genes are important apoptotic factors [32]. Nitrite stress can trigger apoptosis through JNK- and caspase-dependent pathways in mud crabs (*Scylla paramamosain*) [33]. In this study, the increased levels of *JNK*, *NF-κB*, *TNF-α*, *Casp-9,* and *Casp-3* genes revealed that 5 mg/L nitrite stress caused inflammation and apoptosis occurred in the intestine of shrimps. CYP450 and GST are phase I and II detoxification enzymes, respectively [34]. In this study, CYP450 and GST genes showed an up-regulated expression trend after nitrite stress, indicating that detoxification metabolism was activated in response to nitrite-induced cytotoxicity. Consequently, nitrite stress disrupts intestinal homeostasis in shrimps by affecting immunity, inflammation, apoptosis, and detoxification.

### 4.3. Intestinal Energy Metabolism Response of Shrimps to Nitrite Stress

Nutrient metabolism provides energy for organisms. Glycolysis is an important part of glucose metabolism, in which HK, PK, and PDH are key enzymes, whereas LDH is a regulator of anaerobic glycolysis and gluconeogenesis [35,36]. Nitrite stress can affect the activities of HK, PK, and LDH in the hepatopancreas of *L. vannamei* [12]. In this study, *HK*, *PK*, *PDH,* and *LDH* were up-regulated after 5 mg/L nitrite stress, indicating that the intestinal glucose metabolism of shrimps was induced, which may be a positive response of the organism to stress. Lipid metabolism is an important strategy used by aquatic animals to cope with environmental stress. Among them, FAS, SREBP, and ACC catalyze fatty acid synthesis [37], whereas AMPK regulates lipid decomposition and promotes ATP production [38]. In the present study, increased levels of *FAS*, *SREBP*, *ACC,* and *AMPK* indicated that intestinal lipid metabolism was induced in response to stress. The TCA cycle is a hub of nutrient metabolism and is an efficient way to generate energy [39]. In the present study, after 5 mg/L nitrite stress, genes of key enzymes of the TCA cycle in shrimps, such as *MDH*, *CS*, *IDH*, *ODH*, *SDH,* and *FH*, were up-regulated in the shrimp intestine, indicating that the cycle was activated. These results suggest that nitrite stress could disturb the intestinal energy metabolism of the shrimp by affecting the metabolism of the nutrients.

The electron transport chain is the most basic function of mitochondria; it produces ATP to support the life activities of organisms. The electron transport chain comprises a series of transmembrane complexes, including four enzyme complexes, two mobile electron carriers, and one non-respiratory complex [40]. In this study, the increased levels of *NDH*, *SDH*, *CCO*, *COI*, *AtpH,* and *CytC* suggested that the function of the intestinal electron transport chain was activated to produce ATP to prevent nitrite stress.

### 4.4. Intestinal Microbiota Response of Shrimps to Nitrite Stress

A stable intestinal microbiota can provide a biological barrier to the host [41]. Nitrite stress disrupts the intestinal bacterial community by altering host–community interactions in shrimps [21]. Chronic nitrite stress disturbs intestinal microbiota homeostasis in *L. vannamei* [7]. In the present study, nitrite stress slightly increased the intestinal microbial diversity of shrimps, indicating that the microbiota responded positively to stress. Furthermore, the community composition was altered. Additionally, a reduction in Proteobacteria levels and elevated levels of Bacteroidetes, Tenericutes, and Actinobacteria indicated that nitrite stress disturbed the homeostasis of the intestinal bacterial community composition. Furthermore, the metabolisms of “amino sugar and nucleotide sugar”, “histidine”, and “lipoic acid” and the functions of “flavonoid biosynthesis” and “lipid biosynthesis proteins” of intestinal microbiota were elevated after nitrite stress, indicating that the metabolic function of intestinal microbes was affected.

Some beneficial bacteria also fluctuated in abundance. *Alloprevotella*, *Bacteroidales S24-7 group*, *Clostridium*, *Faecalibacterium*, *Romboutsia*, and *Ruminococcaceae* can produce short-chain fatty acids that are beneficial for improving intestinal health [42,43,44,45,46,47]. *Bifidobacterium* and *Lactobacillus* are well-known probiotics [48,49]. *Pseudoalteromonas* enhances the immunity of shrimps [50]. In this study, the decreased levels of *Clostridium sensu stricto 1*, *Faecalibacterium*, *Romboutsia*, and *Ruminococcaceae UCG-010* suggested that nitrite stress influenced the balance of these bacteria involved in producing beneficial substances, which was not detrimental to host health. Intestinal bacteria also respond positively to stress. For example, the abundance of beneficial bacteria such as *Alloprevotella*, *Bifidobacterium*, *Lactobacillus*, *Bacteroidales S24-7 group*, and *Pseudoalteromonas* increased after nitrite stress. These phenomena indicate that nitrite stress interferes with the homeostasis of beneficial substance-producing bacteria in the shrimp intestine.

Harmful bacteria inhabit the intestines of shrimp and can affect their health. *Nautella* often increases in abundance in the intestines of diseased and stressed shrimps [51,52]. Similarly, in this study, the increased levels of *Nautella* indicated that nitrite stress may increase the risk of invasion by harmful bacteria. *Vibrio*, *Photobacterium*, and *Streptococcus* are common opportunistic pathogens in aquaculture [53,54,55]. In this study, the function of “lipopolysaccharide biosynthesis proteins” of intestinal microbes was elevated, suggesting that nitrite stress could induce an elevated risk of bacterial endotoxin. However, the abundances of *Vibrio*, *Photobacterium*, and *Streptococcus* decreased after nitrite stress. We concluded that nitrite stress may alter the environment of intestinal regions inhabited by these pathogens or that the increase in beneficial bacteria may have certain inhibitory effects on these pathogens. However, the specific reasons for this need to be explored further.

## 5. Conclusions

**Figure 8:**
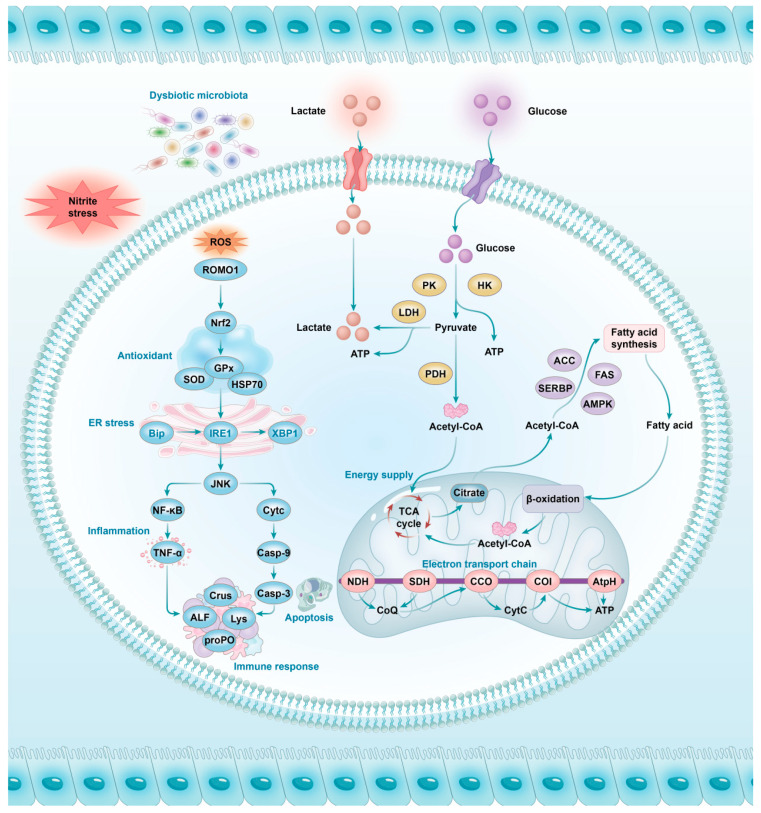
The deduced possible mechanism of the detrimental effects of acute nitrite stress on the intestinal health homeostasis of *L. vannamei*.

**Figure 9:**
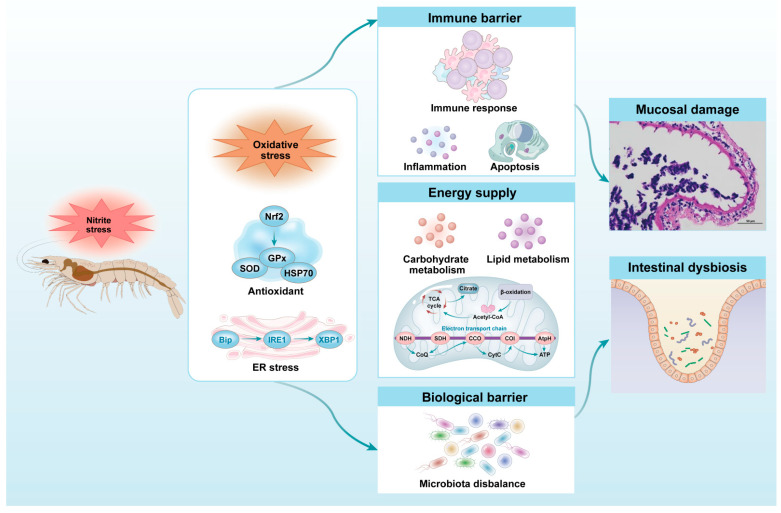
A graphic summary of this study.

This study revealed the harmful effects of acute nitrite stress on the intestinal health of shrimps, and the potential mechanism may be as follows (Figure 8): nitrite stress affects the redox process and induces ER stress by mediating the *Bip*/*IRE1*/*XBP1* pathway, then causes apoptosis and inflammation by activating the *CytC*/*Casp-9*/*Casp-3* and *JNK*/*NF-κB*/*TNF-α* pathways, and further confuses immune homeostasis. The intestine responds to stress by activating *Nrf2* antioxidant signaling and energy metabolism processes. Furthermore, homeostasis of the intestinal microbial community is disturbed, especially for some beneficial and harmful bacteria. This eventually causes mucosal damage and impairs intestinal health (Figure 9). These results contribute to the development of microecological regulation strategies for shrimp intestines to prevent nitrite stress.

## Data Availability

Data is contained within the article and Supplementary Materials.

## Supplementary Materials

The following supporting information can be downloaded at: https://www.mdpi.com/article/10.3390/antiox13111318/s1, Figure S1: The correlation networks of intestinal bacteria at the phylum level; Table S1: Primer sequences used in this study; Table S2: The PERMANOVA analysis of the β-diversity of the intestinal microbiota; Table S3: Changes in the relative abundance of intestinal bacterial phyla of L. vannamei after acute nitrite stress; Table S4: Changes in the relative abundance of intestinal bacterial genera of L. vannamei after acute nitrite stress.
