# Supplementary material for: Effects of Nitrite Stress on the Antioxidant, Immunity, Energy Metabolism, and Microbial Community Status in the Intestine of Litopenaeus vannamei
Source: Antioxidants (Basel). 2024 Oct 29;13(11):1318. doi: 10.3390/antiox13111318 (PMC11591295; doi:10.3390/antiox13111318)
Supplement: Supplementary file 1 [file antioxidants-13-01318-s001.zip › antioxidants-3233375-supplementary.pdf]

Supplementary materials

Table S1 Primer sequences used in this study.

Gene	Forward primer (5’-3’)	Reverse primer (5’-3’)
ROMO1	GCACCGTGGAGTAGGAGAACAAC	TTGCCATGCCGACTGAGAAACC
Nrf2	GATGAGAAGCGAGCCAGAGCG	GCCGTCGGATGTCTCGGATAA
SOD	GACACGACCATTAGCCTGTACGAC	CAGCGTTGCCAGTAGCGAGTG
GPx	TCTGAGCGGCGAGATGGTGTC	CTGGTGGAAGTCCCTGGTGGTC
HSP70	TGTGCCTGCCTACTTCAACGATTC	CGCTCACCGCCAACCTTCTTG
Bip	CCAGCACGACATCCAGTTCTTCC	CCTCCGCAGCAAACACCTTCTC
IRE1	GGTACATTAGGTTCTCGTCCGTCAC	AATTCCTCTGGTGTTGCCTTAGCC
XBP1	CGCCTGCTGAGGATGACCTTATTAC	GCCTACTGGTGATGTGTCCTTAACG
JNK	TCTACCTGGTGATGGAGCTGATGG	CGGCTGAGTGTAAGTGCTTTATCCC
NF-κB	TCTAACCAATCACCACAGCAC	TGGTAAACTCAGTGTTCGGG
TNF-α	TTGGTGGGCAATCACTTGACAGAC	GTGCTGGGCGATATGGAGAAGAC
Casp-3	AGACGGACAGCATACAGGAGGAC	CTCGGCCAAGAAGTGGATGAAGAC
Casp-9	ATGGCTCGTGGTTCATTCAG	CATCAGGGTTGAGACAATACAGG
ALF	GGTGTTCCTGGTGGCACTCT	AGCTCCGTCTCCTCGTTCCT
Crus	TGGTGTAGGTGGCGGTCTTGG	CTTGTGGGCAGTCGAGTATCTTGG
Lys	TCGAGTCGTCCTTCAACACG	TGCAGACGTTCTTGCCGTAG
proPO	CAATGACCAGCAGCGTCTTC	CACGGAAGGAGGCGTATCAT
CYP450	CAACGACACCATCAGGAGCAGAC	CAGCAGCAGGTCCAAGAAGGC
GST	ACGAACACTACGAACAGAAGGATGC	GCCAGGAAGTCGATGTAGGTTAGC
PDH	TCAGCCTCAACCAACTACTACACTC	GCCTCCTTCACACTCAGTACATCC
HK	ACCTGCTGCTGGTTCACGATG	GCTGCTGCCTCCTCCAAGTG
PK	GCCAGACAGTGCCATCTCTACC	TGCCAGCCAGTCACCACAAC
LDH	GATCGGCTCAGGCACCAACC	GCAACATTAACACCAGACCAGACAG
AMPK	GTAGTCCACGATCACCACTCAATGC	CCACCTGCTCCTCCTACTCCATC
SREBP	AGATGGCTGAGATGTTGGTAATGGC	CCCTTGTGGCTCTTCCTCTTTGC
ACC	CGGCAGACAACATCCATACCACAG	GCAACCAGCGAGAGCAGTAACC
FAS	TGCTACTGTGCCTGTTGTGTATGC	CCACCAGAACCTGCGTGAATGAG
CS	GCTCGGTTCCATCCATCCTCTG	ACGCCTTCTGTTGGTGTCCTAAG
IDH	ACGGAGACCAATACAAGGCTACTG	TGAGTGTGCGAAGGAACGGATAG
ODH	GCAAGGCATAATCAGGGCATATCAG	CATAGGAGCGGACTACTGTTCTGG
SDH	TTCCTGGCACTCACTATGACTGTTC	GATGAAGTAGCAGAGACCTCCCAAG
FH	TTGTTCCTCTTCTCGCCTGGTTAC	TCTGACTCCATCTTTGCCACTCTTC
MDH	CTCTTCCACCCAGTTCCCAGATG	GACAACTTACGAGCGGCAATGAC
NDH	CTTCTTGGTTCGGTGCTTGAATGG	AGCAGCCTCTGAAGAGTATTGGTTG
COI	CCCAGATATAGCCTTCCCTCGAATG	GCGTGAGCAATACTGGCAGATAAAG
CCO	ATGCCAGGTGTCCGCTTCAAG	AAGGGTCAACTTGTTCCAGTCTCC
cytC	TCGACGTGTACCTGACCAACCC	TTCGCCTGGCTTCCTCTTCCTC
AtpH	CACCATCATCAACCAGAAGCGATTC	GGAGCAGCATCAGAGGCAGTG
β-actin	TCGCTCCCTCCACCATGAAGATC	CTCCTGCTTGCTGATCCACATCTG
515F	GTGCCAGCMGCCGCGG	
806R		GGACTACHVGGGTWTCTAAT

Table S2 The PERMANOVA analysis of the *β*-diversity of the intestinal microbiota.

Index	Value
F-test	2.0528
R	0.40627
P	0.062

Table S3 Changes in the relative abundance of intestinal bacterial phyla of *L. vannamei* after acute nitrite stress. The same lowercase letters of the data indicate no significant difference ($P > 0.05$), and different letters represent significant difference ($P < 0.05$).

Phylum	CK group	N1 group	N5 group
Proteobacteria	59.48 ± 7.52[a]	49.09 ± 9.63[a]	50.45 ± 22.58[a]
Bacteroidetes	14.69 ± 5.88[a]	27.25 ± 7.91[a]	20.20 ± 10.02[a]
Firmicutes	20.76 ± 9.018[a]	8.95 ± 2.88[a]	19.61 ± 14.89[a]
Tenericutes	2.13 ± 0.60[a]	8.81 ± 3.38[a]	3.28 ± 2.27[a]
Cyanobacteria	0.401 ± 0.07[a]	0.35 ± 0.09[a]	0.14 ± 0.10[a]
Actinobacteria	0.95 ± 0.52[a]	4.24 ± 1.47[a]	4.54 ± 2.20[a]
Fusobacteria	0.22 ± 0.13[a]	0.10 ± 0.04[a]	0.09 ± 0.06[a]
Planctomycetes	0.07 ± 0.02[a]	0.34 ± 0.11[a]	0.44 ± 0.32[a]
Verrucomicrobia	0.11 ± 0.04[a]	0.08 ± 0.03[a]	0.05 ± 0.03[a]
Acidobacteria	0.06 ± 0.01[a]	0.26 ± 0.22[a]	0.09 ± 0.07[a]
Others	1.12 ± 0.96[a]	0.53 ± 0.17[a]	1.10 ± 0.86[a]

Table S4 Changes in the relative abundance of intestinal bacterial genera of *L. vannamei* after acute nitrite stress. The same lowercase letters of the data indicate no significant difference ($P > 0.05$), and different letters represent significant difference ($P < 0.05$).

Genus	CK group	N1 group	N5 group
Lactobacillus	4.68 ± 0.63[a]	4.74 ± 1.49[a]	10.15 ± 8.0[a]
Candidatus Bacilloplasma	1.64 ± 0.25[a]	8.74 ± 3.38[a]	3.20 ± 2.26[a]
Bacteroidales S24-7 group	0.52 ± 0.07[a]	0.98 ± 0.38[a]	10.85 ± 10.14[a]
Bifidobacterium	0.08 ± 0.01[a]	0.38 ± 0.26[a]	0.20 ± 0.18[a]
Pseudoalteromonas	0.08 ± 0.03[a]	9.45 ± 2.39[a]	8.84 ± 8.83[a]
Demequina	0.03 ± 0.01[a]	3.05 ± 1.29[a]	2.78 ± 2.73[a]
Alloprevotella	0.02 ± 0.01[a]	0.06 ± 0.02[a]	2.44 ± 2.38[a]
Bacillus	0.01 ± 0.00[a]	0.05 ± 0.01[b]	0.01 ± 0.01[a]
Spongiimonas	10.50 ± 5.60[a]	0.05 ± 0.01[a]	2.94 ± 2.91[a]
Romboutsia	1.10 ± 1.07[a]	0.07 ± 0.03[a]	0.05 ± 0.04[a]
Faecalibacterium	0.92 ± 0.90[a]	0.03 ± 0.01[a]	0.02 ± 0.02[a]
Clostridium sensu stricto 1	0.72 ± 0.55[a]	0.41 ± 0.17[a]	0.07 ± 0.03[a]
Rikenellaceae RC9 gut group	0.12 ± 0.01[b]	0.04 ± 0.02[a]	0.03 ± 0.02[a]
Ruminococcaceae UCG-010	0.07 ± 0.02[b]	0.01 ± 0.00[a]	0.02 ± 0.01[a]
Mogibacterium	0.01 ± 0.00[b]	0.00 ± 0.00[a]	0.00 ± 0.00[a]
Nautella	0.13 ± 0.06[a]	0.39 ± 0.15[a]	2.24 ± 2.19[a]
Photobacterium	25.91 ± 2.23[a]	8.00 ± 2.52[a]	14.55 ± 13.04[a]
Vibrio	17.64 ± 5.77[a]	3.35 ± 2.03[a]	8.80 ± 4.42[a]
Streptococcus	1.07 ± 0.47[a]	0.38 ± 0.12[a]	0.25 ± 0.14[a]

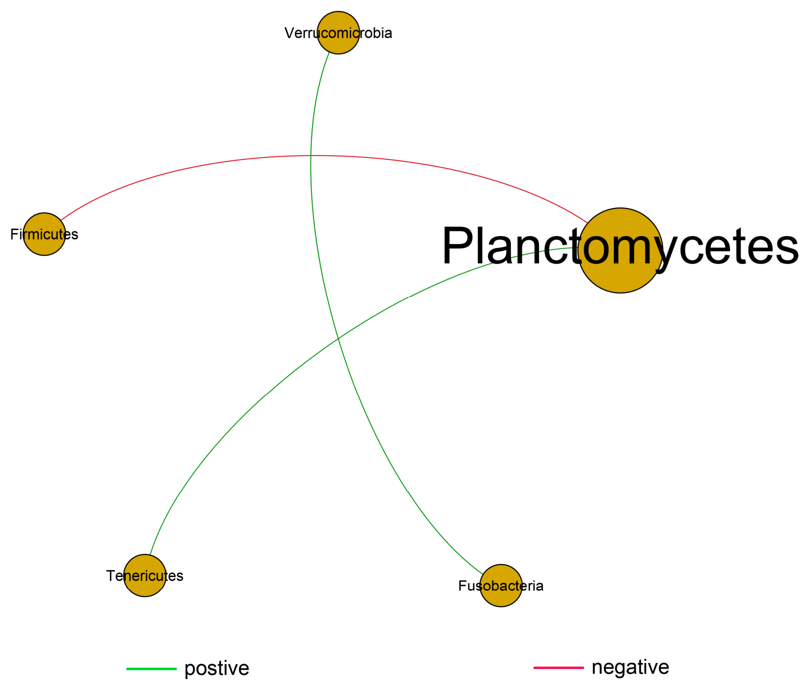

Figure S1. The Correlation networks of intestinal bacterial at the phylum level. The red line indicates negative correlation and the green line indicates positive correlation.
